# mSphere of Influence: Drivers of Host-Associated Microbial Community Structure and Change

**DOI:** 10.1128/mSphere.00248-21

**Published:** 2021-04-07

**Authors:** Vanessa L. Hale

**Affiliations:** a Veterinary Preventive Medicine, Ohio State University College of Veterinary Medicine, Columbus, Ohio, USA

**Keywords:** diet, evolution, fiber, gut microbiome, gut microbiota

## Abstract

Vanessa L. Hale studies the role of the microbiome in disease susceptibility in animal and human health. In this mSphere of Influence article, she reflects on how the papers “Evolution of mammals and their gut microbes” (R. E. Ley, M. Hamady, C. Lozupone, P. J. Turnbaugh, et al., Science 320:1647–1651, 2008, https://doi.org/10.1126/science.1155725) and “A dietary fiber-deprived gut microbiota degrades the colonic mucus barrier and enhances pathogen susceptibility” (M. S. Desai, A. M. Seekatz, N. M. Koropatkin, N. Kamada, et al., Cell 167:1339–1353.e21, 2016, https://doi.org/10.1016/j.cell.2016.10.043) have provided a foundation for studying drivers of gut microbial structure and change across host species in the context of evolution and disease risk.

## COMMENTARY

What are the ultimate and proximate drivers of host-associated microbial community structure and change? Work across disciplines from medicine to agriculture to anthropology actively seeks to answer this question in part by determining how these communities can be manipulated to promote health and prevent or treat disease. Two papers that set the stage for current work and profoundly shaped my own thinking include Ley et al. (2008), “Evolution of mammals and their gut microbes,” and Desai et al. (2016), “A dietary fiber-deprived gut microbiota degrades the colonic mucus barrier and enhances pathogen susceptibility” (1, 2). Ley’s paper (from J. I. Gordon’s research group) framed gut microbial communities on a long-term evolutionary time scale and demonstrated that host diet (herbivore, omnivore, carnivore) and phylogeny shape gut microbial diversity between hosts. Desai’s work (from E. C. Martens’ research group) illustrated microbial community change within hosts on a short-term, daily time scale and how diet-microbe interactions impact host health and enteric pathogen susceptibility. Both studies examine factors that shape gut microbial communities but on two very different time scales.

Ley et al. (2008) compared the gut microbial communities of 60 animal species, including humans, and evaluated the influence of diet, host taxonomic order, and sample provenance on gut microbial composition and diversity across host species. The authors hypothesized that gut microbiota play a critical role in the acquisition of new dietary niches, which can ultimately lead to speciation. They identified similarities in microbial communities based on host diet and phylogeny, and they made several unexpected observations: foregut and hindgut fermenting herbivores had similar diets but distinct microbial communities; herbivorous pandas, with simple guts, had microbial communities similar to other simple-gutted carnivores despite their dietary differences; and humans had microbial communities similar to other omnivorous species. This work provided a foundation for future studies on the gut microbiome and evolution/coevolution, diet, fiber consumption, and host physiology (3, 4), and it paved a way for thinking about translational models for microbiome research. Desai et al. (2016) evaluated many of the same factors—diet, fiber consumption, host physiology—in a series of experiments that examined gut microbial community function and change over time in response to differing dietary regimens. In their germfree mouse model inoculated with a synthetic human gut microbiota, fiber-free diets or alternating fiber-rich/fiber-free diets resulted in an increased abundance of mucin-degrading bacteria, an increase in microbial mucus-targeting transcripts, a thinner mucus layer in the mouse gut, a decreased colon length, and an increase in gut inflammatory markers. These changes increased susceptibility to the enteric pathogen Citrobacter rodentium, which is a mouse model for enteropathogenic Escherichia coli. This work is an elegant example, among others (5, 6), revealing that “one microbe” does not automatically equal “one disease”; rather, there is a rich interaction between microbial communities, diet, and host mucin production, which affects disease susceptibility and pathogenesis.

The question at the core of my research is: how do host-associated microbial communities shape disease susceptibility in animals? I study animal models of diseases including clostridial enteritis and bladder cancer that have translational relevance to human health. From a 30,000-foot view, this requires me to consider how similarities or differences in host taxa broadly impact microbial community structure, host-microbe relationships, and disease risks between species. At a finer scale, I evaluate how microbes interact within communities and hosts, and in response to external factors like diet or xenobiotics. Both the Ley and Desai papers expanded my scientific horizons by revealing how we could study microbial communities from ultimate and proximate perspectives. The Ley et al. paper applied recently developed tools (UniFrac) for comparing microbial communities (7, 8), while the Desai et al. paper applied a tour de force of both established and novel methodologies for studying the microbiota—ranging from culture and histopathology, to gnotobiotic mouse models, to designing a synthetic microbial community, to laser capture microdissection of a colon section for separate analyses of lumen and mucus layers, to transcriptomics and metabolomics, to alcian blue and MUC2 staining for measuring mucin thickness. At different levels, both of these papers captured my scientific imagination and showed me how I could work at the intersection of animal and human health studying the effects of microbial communities on disease susceptibility.
